# Editorial: Multidisciplinary aspects and performance in racket sports, volume II

**DOI:** 10.3389/fpsyg.2025.1632789

**Published:** 2025-06-13

**Authors:** Rafael Martínez-Gallego, Bernardino Javier Sánchez-Alcaraz Martínez, Goran Vuckovic, Jesús Ramón-Llin

**Affiliations:** ^1^Research Group on Sports Technique and Tactics, University of Valencia, Valencia, Spain; ^2^Department of Physical Activity and Sport, Faculty of Sport Science, University of Murcia, Murcia, Spain; ^3^Faculty of Sports, University of Ljubljana, Ljubljana, Slovenia

**Keywords:** racket sports, performance, psychology, skill acquisition, training, coaching

Racket sports continue to captivate athletes, coaches, researchers, and practitioners around the world, driven by their unique combination of dynamic physical demands, technical skill, tactical decision-making, and psychological performance. Building on the contributions of prior research, this second volume of the Research Topic “*Multidisciplinary aspects and performance in racket sports, volume II”* offers new perspectives and deepens our understanding of the diverse factors that influence performance, development, and wellbeing across multiple racket disciplines.

One prominent theme in this volume is the psychological dimension of racket sports, encompassing both the enhancement of wellbeing and the management of competitive stress. Sun et al. explore the positive impact of tennis on university students, showing how participation can reduce depressive symptoms and promote prosocial behavior, with social support playing a mediating role. In the competitive sphere, Conde-Ripoll et al. examine pre- and post-competition anxiety and self-confidence in elite male padel players, revealing links to technical and tactical performance and offering guidance for psychological preparation. Complementing this focus on performance under pressure, Mizuno and Masaki investigate the effects of left-hand contraction on tennis serve performance, suggesting a simple and practical strategy to reduce choking risk and enhance execution during high-pressure moments.

Skill acquisition and pedagogical innovation are equally central to this Research Topic. Morales-Campo et al. introduce Touchtennis as an engaging alternative for physical education, aiming to broaden participation and enhance accessibility among schoolchildren. In a related study, Ortega-Zayas et al. develop and validate a questionnaire designed to assess the quality of table tennis instruction in educational settings, offering educators a practical assessment tool. Expanding on these themes, Piquer-Piquer et al. present a scoping review on equipment modifications for novice tennis players. Their synthesis highlights how scaled equipment supports skill development, supports performance and enhances enjoyment—while also addressing biomechanical and coaching considerations.

Technical skill development and anticipation training are further examined as key elements for success in racket sports. Triolet and Benguigui investigate the efficacy of opponent-specific, implicit anticipation training in expert tennis players, demonstrating improvements in predictive accuracy. At the developmental level, Diler et al. propose a structured tool to assess groundstroke technique in preadolescent players, providing coaches with objective criteria to guide early technical training.

Physical conditioning and performance assessment remain critical to optimizing athletic potential. Wang and Xu evaluate the effects of different frequencies of upper limb plyometric training on strength, power, and serve velocity in young male tennis players, offering evidence-based recommendations for training design. In a broader context, Morais et al. assess the impact of a 6-week court-based training program on the International Tennis Number (ITN) and various physical fitness indicators in youth players, identifying key predictors of the ITN relevant for talent identification and progression. Additionally, Winata et al. present a systematic review of match-play data in badminton, highlighting specific demands across the five competitive categories—insights that are crucial for guiding training and evaluation approaches.

In summary, the contributions in “*Multidisciplinary aspects and performance in racket sports, volume II”* reflect the growing complexity and breadth of this field. Spanning psychology, pedagogy, biomechanics, motor learning, and physical conditioning, these studies offer relevant insights and tools for researchers, practitioners, educators, and coaches. We are confident that this Research Topic will not only inform current practices but also inspire continued exploration and innovation in the dynamic world of racket sports.

